# Long-Term Outcome of Immediate Versus Postponed Intervention in Patients With Infected Necrotizing Pancreatitis (POINTER): Multicenter Randomized Trial

**DOI:** 10.1097/SLA.0000000000006001

**Published:** 2023-07-17

**Authors:** Charlotte L. Van Veldhuisen, Noor J. Sissingh, Lotte Boxhoorn, Sven M. van Dijk, Janneke van Grinsven, Robert C. Verdonk, Marja A. Boermeester, Stefan A.W. Bouwense, Marco J. Bruno, Vincent C. Cappendijk, Peter van Duijvendijk, Casper H J. van Eijck, Paul Fockens, Harry van Goor, Muhammed Hadithi, Jan Willem Haveman, Maarten A.J.M. Jacobs, Jeroen M. Jansen, Marnix P.M. Kop, Eric R. Manusama, J. Sven D. Mieog, I. Quintus Molenaar, Vincent B. Nieuwenhuijs, Alexander C. Poen, Jan-Werner Poley, Rutger Quispel, Tessa E.H. Römkens, Matthijs P. Schwartz, Tom C. Seerden, Marcel G.W. Dijkgraaf, Martijn W.J. Stommel, Jan Willem A. Straathof, Niels G. Venneman, Rogier P. Voermans, Jeanin E. van Hooft, Hjalmar C. van Santvoort, Marc G. Besselink

**Affiliations:** * Department of Surgery, Amsterdam UMC, University of Amsterdam, Amsterdam, The Netherlands; † Amsterdam Gastroenterology Endocrinology Metabolism, The Netherlands; ‡ Department of Research and Development, St. Antonius Hospital, Nieuwegein, The Netherlands; § Department of Gastroenterology and Hepatology, Leiden University Medical Centre, Leiden, The Netherlands; ∥ Department of Gastroenterology and Hepatology, Amsterdam UMC, location University of Amsterdam, Amsterdam, The Netherlands; ¶ Department of Gastroenterology and Hepatology, St. Antonius Hospital, Nieuwegein, The Netherlands; # Department of Surgery, Maastricht University Medical Centre+, Maastricht, The Netherlands; ** Department of Gastroenterology and Hepatology, Erasmus Medical Centre, Rotterdam, The Netherlands; †† Department of Radiology, Jeroen Bosch Hospital, Den Bosch, The Netherlands; ‡‡ Department of Surgery, Gelre Hospital, Apeldoorn, The Netherlands; §§ Department of Surgery, Erasmus Medical Centre, Rotterdam, The Netherlands; ∥∥ Department of Surgery, Radboud University Medical Centre, Nijmegen, The Netherlands; ¶¶ Department of Gastroenterology and Hepatology, Maasstad Hospital, Rotterdam, The Netherlands; ## Department of Surgery, University Medical Centre Groningen, University of Groningen, Groningen, The Netherlands; *** Department of Gastroenterology and Hepatology, Amsterdam UMC, location Vrije Universiteit Amsterdam, Amsterdam, The Netherlands; ††† Department of Gastroenterology and Hepatology, OLVG, Amsterdam, The Netherlands; ‡‡‡ Department of Radiology, Amsterdam UMC, location University of Amsterdam, Amsterdam, The Netherlands; §§§ Department of Surgery, Medical Centre Leeuwarden, Leeuwarden, The Netherlands; ∥∥∥ Department of Surgery, Leiden University Medical Centre, Leiden, The Netherlands; ¶¶¶ Department of Surgery, University Medical Centre Utrecht, Utrecht, The Netherlands; ### Department of Surgery, Isala Clinics, Zwolle, The Netherlands; **** Department of Gastroenterology and Hepatology, Isala Clinics, Zwolle, The Netherlands; †††† Department of Gastroenterology and Hepatology, Maastricht University Medical Centre+, Maastricht, The Netherlands; ‡‡‡‡ Department of Gastroenterology and Hepatology, Reinier de Graaf Group, Delft, The Netherlands; §§§§ Department of Gastroenterology and Hepatology, Jeroen Bosch Hospital, Den Bosch, The Netherlands; ∥∥∥∥ Department of Gastroenterology and Hepatology, Meander Medical Centre, Amersfoort, The Netherlands; ¶¶¶¶ Department of Gastroenterology and Hepatology, Amphia Hospital, Breda, The Netherlands; #### Department of Epidemiology and Data Science, Amsterdam UMC, location University of Amsterdam, Amsterdam Public Health, Amsterdam, The Netherlands; ***** Department of Gastroenterology and Hepatology, Máxima Medical Centre, Veldhoven, The Netherlands; ††††† Department of Gastroenterology and Hepatology, Medisch Spectrum Twente, Enschede, The Netherlands; ‡‡‡‡‡ Department of Surgery, St. Antonius Hospital, Nieuwegein, The Netherlands; §§§§§ Department of Surgery, University Medical Center Utrecht, The Netherlands

**Keywords:** antibiotics, clinical outcomes, delayed, drainage, early, infected necrotizing pancreatitis, necrosectomy, timing of intervention

## Abstract

**Objective:**

To compare the long-term outcomes of immediate drainage versus the postponed-drainage approach in patients with infected necrotizing pancreatitis.

**Background:**

In the randomized POINTER trial, patients assigned to the postponed-drainage approach using antibiotic treatment required fewer interventions, as compared with immediate drainage, and over a third were treated without any intervention.

**Methods:**

Clinical data of those patients alive after the initial 6-month follow-up were re-evaluated. The primary outcome was a composite of death and major complications.

**Results:**

Out of 104 patients, 88 were re-evaluated with a median follow-up of 51 months. After the initial 6-month follow-up, the primary outcome occurred in 7 of 47 patients (15%) in the immediate-drainage group and 7 of 41 patients (17%) in the postponed-drainage group (RR 0.87, 95% CI 0.33–2.28; *P*=0.78). Additional drainage procedures were performed in 7 patients (15%) versus 3 patients (7%) (RR 2.03; 95% CI 0.56–7.37; *P*=0.34). The median number of additional interventions was 0 (IQR 0–0) in both groups (*P*=0.028). In the total follow-up, the median number of interventions was higher in the immediate-drainage group than in the postponed-drainage group (4 vs. 1, *P*=0.001). Eventually, 14 of 15 patients (93%) in the postponed-drainage group who were successfully treated in the initial 6-month follow-up with antibiotics and without any intervention remained without intervention. At the end of follow-up, pancreatic function and quality of life were similar.

**Conclusions:**

Also, during long-term follow-up, a postponed-drainage approach using antibiotics in patients with infected necrotizing pancreatitis results in fewer interventions as compared with immediate drainage and should therefore be the preferred approach.

**Trial registration:**

ISRCTN33682933

Acute pancreatitis mostly runs a mild clinical course, but 20% of patients develop severe pancreatitis with necrosis.^1–4^ Secondary infection of pancreatic and peripancreatic necrosis puts these patients at risk of significant morbidity and 10% to 39% mortality.^5^ Several randomized studies have attempted to optimize the treatment of patients with infected necrotizing pancreatitis.^6–11^ Besides antibiotic treatment, the minimally invasive step-up approach, with catheter drainage of the infected necrotic collection as the first step, followed by minimally invasive necrosectomy when needed, is the current standard treatment strategy. However, the optimal timing of drainage in infected necrotizing pancreatitis remains unknown and varies widely in current practice.^12–14^

The recent multicenter randomized POINTER trial compared immediate catheter drainage within 24 hours after diagnosing infected pancreatic necrosis with postponed catheter drainage.^11^ At 6-month follow-up, immediate drainage was not superior to postponed drainage regarding complications. In fact, the postponed-drainage approach significantly reduced the number of invasive interventions, both catheter drainage and necrosectomy. Some 19 patients (39%) assigned to the postponed-drainage group did not require any intervention because their clinical condition improved with antibiotic treatment only; 17 of these patients (35%) survived. The question remains whether these relative benefits of the postponed-drainage approach persist after the initial 6-month follow-up. Some have argued that infected (peri) pancreatic necrotic collections, which are initially treated conservatively with antibiotics, could lead to persistent complications requiring intervention and ultimately causing mortality during longer follow-up.

Therefore, the current study evaluates new events beyond the initial 6-month follow-up on long-term clinical outcomes of patients enrolled in the POINTER trial.

## METHODS

### Study Design

Between August 2015 and October 2019, a total of 104 patients with infected necrotizing pancreatitis were enrolled in the multicenter randomized POINTER (Postponed or Immediate Drainage of Infected Necrotizing Pancreatitis) trial.^15,16^ The study was conducted in 22 Dutch hospitals collaborating with the Dutch Pancreatitis Study Group (DPSG). Infected necrosis was defined as either a positive fine-needle aspiration (FNA) culture, presence of gas in (peri)pancreatic necrosis on contrast-enhanced computed tomography, and after 14 days of onset, clinical signs of infection were also considered to be diagnostic if other causes of infections were ruled out. Clinical signs of infection were defined as: persistent (multiple) organ failure or the presence of 2 of 3 elevated inflammatory parameters (temperature >38.5, C-reactive protein levels or leukocyte count) for 3 consecutive days. Patients were randomly assigned to immediate catheter drainage (55 patients) or postponed catheter drainage (49 patients). The study protocol of the current investigator-initiated long-term follow-up study was approved by the institutional review board of the Amsterdam UMC. All authors had access to the study data and reviewed and approved the final version of the manuscript. The study was conducted in accordance with the principles of the Declaration of Helsinki and reported according to the STROBE Checklist (Supplementary Table S1, Supplemental Digital Content 1, http://links.lww.com/SLA/E733).^17^

### Long-Term Follow-Up Protocol

Surviving patients from the POINTER trial were informed about the study by telephone and subsequently invited to participate. Written informed consent was obtained from all patients with the exception of deceased patients. Eligible patients were evaluated until June 2022, following the initial POINTER study, which had a 6-month follow-up. Clinical data regarding death, complications, interventions (ie, drainage and necrosectomy procedures), readmission and disease course was retrieved retrospectively from medical records. Interventional procedures related to disconnected pancreatic duct syndrome were also recorded. Additional data were collected by a telephone conversation with patients or family members by the study coordinators (C.vV. and N.S.). The choice of treatment (ie, type and timing of interventions) was left to the treating physician, and no particular criteria were formulated to guide the decisions of the physicians. For data collection, online database software (Castor EDC, Amsterdam, the Netherlands) was used.

### Outcomes

The primary outcome was a composite of death and major complications (ie, new-onset (multiple) organ failure, bleeding requiring intervention, perforation of a visceral organ requiring intervention or enterocutaneous fistula, similar to other trials and follow-up studies performed by our group.^6,18^ This primary outcome differed from the original primary outcome (ie Comprehensive Complication Index [CCI]) because CCI would be less relevant during follow-up because this tool was developed to assess short-term complications.^19–21^ The primary outcome was selected based on the hypothesis that residual (peri)pancreatic necrotic collections, especially in the postponed treatment group, could require new interventions and ultimately cause mortality during longer follow-up. In accordance with the initial study, secondary outcomes included individual major complications, incisional hernia, pancreaticocutaenous fistula, wound infection, interventions, the total length of intensive care, and hospital stay related to pancreatitis length. In addition, the occurrence of recurrent acute pancreatitis and chronic pancreatitis was assessed. Furthermore, we evaluated exocrine and endocrine pancreatic function based on a questionnaire and quality of life measured with the Medical Outcomes Study 36-Item Short-Form General Health Survey (SF-36).^22^ Outcomes were assessed for the period after the trial’s initial 6-month follow-up until the end of long-term follow-up (‘new events after the initial 6-month follow-up’) for all patients who were still alive after the initial 6-month follow-up. Separately, all events between the time of randomization and the end of long-term follow-up (‘total follow-up’) were reported for all patients, including patients who died in the initial 6-month follow-up, with the exception of patients who declined to participate in this follow-up study. This will provide a complete overview and accurate comparison between the 2 different treatment groups.

### Definitions

All definitions were according to the initial POINTER trial and are explained in detail in Supplementary Table S2, Supplemental Digital Content 1, http://links.lww.com/SLA/E733. Patients were considered to have endocrine pancreatic insufficiency in case of use of diabetes medication (ie, oral medication or insulin therapy), not used at the time of randomization. Exocrine pancreatic insufficiency was defined as the use of pancreatic enzymes not used at the time of randomization. We considered successful treatment with antibiotics only if patients survived the initial 6-month follow-up and were treated without any intervention during total follow-up. The diagnosis of disconnected duct was based either on radiological confirmation or on an amylase level in external drain fluid of 3 times the upper limit of normal amylase level. The follow-up period was defined as the time between randomization and the date of data entry in surviving patients or the date of death in deceased patients.

### Statistical Analysis

The analysis was performed according to the intention-to-treat principle. Outcome measures are expressed as means±SD or as medians with interquartile ranges (IQR), depending on the distributional properties. Categorical data are presented as counts and proportions. For normally distributed continuous data, statistical significance was assessed using the Student’s-*t-*test. For non-normally distributed continuous data, the Mann-Whitney *U* test was performed. For categorical data, Fisher’s exact test was performed. Sensitivity analyses excluding patients in whom the diagnosis was based on FNA and radiographic appearance were performed. Results are expressed as relative risks (RRs) with corresponding 95% confidence intervals (CI). All reported *P* values are two-sided, and a *P* value of less than 0.05 was considered statistically significant. *P* values were not adjusted for multiple testing. All statistical analyses were conducted with IBM Statistic SPSS 26.0.

### Funding

This research did not receive any specific funding.

## RESULTS

**FIGURE 1:**
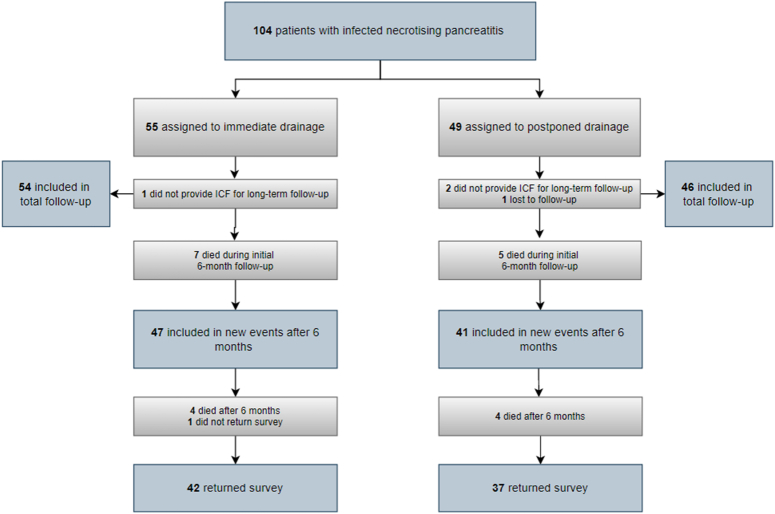
Trial Profile.

Overall, 104 patients with infected necrotizing pancreatitis were randomized in the initial POINTER trial. As shown in Fig. 1, 12 of 104 patients died during the initial 6-month follow-up; 7 patients in the immediate-drainage group versus 5 patients in the postponed-drainage group. Of the 92 surviving patients, 4 patients (who were all still alive) did not consent to participate in the current long-term follow-up study, leaving 88 patients (47 patients in the immediate-drainage group and 41 patients in the postponed-drainage group) to be included in the analysis ‘new events after the initial 6-month follow-up’. These 88 patients, together with the 12 patients who died in the initial 6-month follow-up, were included in the ‘total follow-up’ analysis, resulting in a total of 100 patients (54 in the immediate-drainage group and 46 in the postponed-drainage group). At the end of the long-term follow-up, questionnaires were obtained from 79 patients (42 patients in the immediate-drainage group and 37 patients in the postponed-drainage group). Baseline characteristics were similar between the 2 groups (Supplementary Table S3, Supplemental Digital Content 1, http://links.lww.com/SLA/E733).^11^ The total follow-up was 51 months (IQR 31) (50 months (IQR 32) in the immediate-drainage group and 51 months (IQR 29) in the postponed-drainage group) and did not statistically differ among groups (*P*=0·91).

### New Events After the Initial 6-Month Follow-up

After the initial 6-month follow-up, the composite primary outcome of death and major complications occurred in 7/47 patients (15%) in the immediate-drainage group and 7/41 patients (17%) in the postponed-drainage group (RR 0·87; 95% CI 0·33–2·28; *P*=0·78) (Table 1). Death occurred in 4 patients in the immediate-drainage group (9%) and in 4 patients in the postponed-drainage group (10%) (RR 0·87; 95% CI 0·23–3·27; *P*=1·00). Two deaths in the immediate-drainage group were directly related to pancreatitis, whereas none of the deaths in the postponed-drainage group (Supplementary Table S4, Supplemental Digital Content 1, http://links.lww.com/SLA/E733). No significant differences were found in the individual components of major complications, including new-onset organ failure (9% in the immediate-drainage group and 5% in the postponed-drainage group; RR 1·75; 95% CI 0·34–9·04; *P*=0·68), multiple new-onset organ failure (2% and 0%, respectively; *P*=1·00), bleeding (2% and 0%, respectively; *P*=1·00), and perforation of a visceral organ or enterocutaneous fistula (2% and 2%, respectively; RR 0·87; 95% CI 0·06–13·51; *P*=1·00). The incidence of other outcomes, including incisional hernia (4% and 2%, respectively; RR 2·86; 95% CI 0·32–25·72; *P*=0·54), pancreaticocutaenous fistula (2% and 0%, respectively; *P*=1·00), and wound infection (2% and 5%, respectively; RR 0·44; 95% CI 0·04–4·64; *P*=0·60), did not differ significantly.

**TABLE 1:** Primary and Secondary Outcomes^*^

	New events after the initial 6-month follow-up (excluding events as initially reported in the POINTER trial)				Total follow-up^†^ (Time between randomization and the end of long-term follow-up)			
Outcome	Immediate drainage (n=47)	Postponed drainage (n=41)	Relative risk (95% CI)	P	Immediate drainage (n=54)	Postponed drainage (n=46)	Relative risk (95% CI)	P
Primary outcomes, n (%)								
Major complications or death	7 (15)	7 (17)	0.87 (0.33–2.28)	0.78	26 (48)	21 (46)	1.06 (0.69–1.60)	0.80
Secondary outcomes, n (%)^‡^								
Death	4 (9)	4 (10)	0.87 (0.23–3.27)	1.00	11 (20)	9 (20)	1.04 (0.47–2.29)	0.92
New-onset organ failure	4 (9)	2 (5)	1.75 (0.34–9.04)	0.68	17 (31)	12 (26)	1.21 (0.65–2.26)	0.55
Pulmonary	3 (6)	2 (5)	1.31 (0.23–7.45)	1.00	8 (15)	10 (22)	0.68 (0.29–1.58)	0.37
Cardiovascular	3 (6)	1 (2)	2.62 (0.28–24.19)	0.62	13 (24)	10 (22)	1.11 (0.54–2.29)	0.78
Renal	0	0	−	−	3 (6)	4 (9)	0.64 (0.15–2.71)	0.70
Multiple new-onset organ failure	1 (2)	0	−	1.00	5 (9)	8 (17)	0.53 (0.19–1.52)	0.23
Bleeding requiring intervention	1 (2)	0	−	1.00	8 (15)	10 (22)	0.68 (0.29–1.58)	0.37
Perforation of a visceral organ or enterocutaneous fistula	1 (2)	1 (2)	0.87 (0.06–13.51)	1.00	5 (9)	5 (11)	0.85 (0.26–2.76)	1.00
Other outcomes, n (%)								
Incisional hernia	2 (4)	1 (2)	2.86 (0.32–25.72)	0.54	2 (4)	1 (2)	1.70 (0.16–18.2)	1.00
Pancreaticocutaneous fistula	1 (2)	0	−	1.00	7 (13)	4 (9)	1.49 (0.47–4.77)	0.50
Wound infection	1 (2)	2 (5)	0.44 (0.04–4.64)	0.60	1 (2)	3 (7)	0.28 (0.03–2.64)	0.33
Recurrent acute pancreatitis	7 (15)	5 (10)	1.53 (0.48–4.85)	0.47	NA	NA	NA	NA
Chronic pancreatitis	5 (12)	2 (5)	2.18 (0.45–10.6)	0.44	NA	NA	NA	NA

* Multiple events in the same patient were scored as one outcome.

† 4 patients (of the originally 104 included patients) from the POINTER trial did not consent to participate in this follow-up study and were therefore missing in the total follow-up analysis.

‡ Individual components of the composite primary outcome.

Data are presented as n (%).

Recurrent acute pancreatitis and chronic pancreatitis occurred in 7 patients (15%) and 5 patients (11%) in the immediate-drainage group versus 5 patients (12%) and 2 patients (5%) in the postponed-drainage group (RR 1·53; 95% 0·48–4·85; *P*=0·47; RR 2·18; 95% 0·45–10·6; *P*=0·44), respectively.

One or more drainage procedures were required in 7 patients (15%) in the immediate-drainage group versus 3 patients (7%) in the postponed-drainage group (RR 2·03; 95% CI 0·56–7·37; *P*=0·33) after the initial 6-month follow-up; of which 1 was initially treated with antibiotics alone. Signs of a disrupted or disconnected pancreatic duct were present in 3 of those patients (30%). No patient in both groups needed a necrosectomy after the initial 6-month follow-up. The median number of drainage procedures and necrosectomies was 0 [IQR 0] in both groups (*P*=0·28). More details regarding interventions are given in Supplementary Table S5, Supplemental Digital Content 1, http://links.lww.com/SLA/E733. The median length of intensive care stay was 0 days [IQR 0] in both groups (*P*=0·69), and hospital stay was 0 days [IQR 16] in the immediate-drainage group and 2 [IQR 5] in the postponed-drainage group (*P*=0·09), respectively. Results of the sensitivity analyses are provided in Supplementary Table S6, Supplemental Digital Content 1, http://links.lww.com/SLA/E733.

### Total follow-up

In the total follow-up, the composite primary outcome of death and major complications occurred in 26/54 patients (48%) in the immediate-drainage group and in 21/46 patients (46%) in the postponed-drainage group (RR 1·06; 95% CI 0·69–1·60; *P*=0·80) (Table 1). Death occurred in 11 patients (20%) and 9 patients (20%) in the immediate-drainage group and postponed-drainage group, respectively. No differences were found in the individual components of major complications.

All 54 patients (100%) in the immediate-drainage group underwent catheter drainage in the total follow-up, whereas 30 patients (65%) in the postponed-drainage group (RR 1·53; 95% CI 1·24–1·89; *P*<0·0001) (Table 2). Necrosectomy was performed in 28 patients (52%) in the immediate-drainage group versus 11 patients (24%) in the postponed-drainage group (RR 2.17; 95% CI 1.22–3.86; *P*=0·001). Patients in the postponed-drainage group required fewer catheter drainages (1 [IQR 3] versus 3 [IQR 4]; *P*=0·00) and necrosectomies (1 [IQR 1] versus 2 [IQR 1]; *P*=0·01) compared with patients in the immediate-drainage group. The median number of surgical, endoscopic, and radiologic interventions (catheter drainage and necrosectomy) was 4 [IRQ 5] in the immediate-drainage group versus 1 [IQR 6] in the postponed-drainage group (*P*=0.001).

**TABLE 2:** Interventions and Health Care Utilization^*^

	New events after the initial 6-month follow-up (excluding events as initially reported in the POINTER trial)				Total follow-up^†^ (Time between randomization and the end of long-term follow-up)			
Outcome	Immediate drainage (n=47)	Postponed drainage (n=41)	Relative risk (95% CI)	P	Immediate drainage (n=54)	Postponed drainage (n=46)	Relative risk (95% CI)	P
Catheter Drainage, n (%)	7 (15)	3 (7)	2.03 (0.56–7.37)	0.33	54 (100)	30 (65)	1.53 (1.24–1.89)	0.000
Necrosectomy, n (%)	0	0	−	−	28 (52)	11 (24)	2.17 (1.22–3.86)	0.004
Median total surgical, endoscopic, and radiologic interventions for infected necrosis (IQR), n	0 (0-0)	0 (0–0)	−	0.28	4 (2–7)	1 (0–6)	−	0.001
Median total drainage procedures (IQR), n	0 (0–0)	0 (0–0)	−	0.28	3 (1–5)	1 (0–3)	−	0.000
No. of drainage procedures (%), n (%)								
0	40 (85)	38 (93)	−	−	0	16 (35)	−	−
1	6 (13)	2 (5)	−	−	19 (35)	16 (35)	−	−
2	0	0	−	−	6 (11)	0	−	−
≥3	1 (2)	1 (2)	−	−	29 (54)	14 (30)	−	−
Median total necrosectomies (IQR), n	0 (0–0)	0 (0–0)	−	−	1 (0–1)	0 (0–0)	−	0.01
No. of necrosectomies, n (%)								
0	47 (100)	41 (100)	−	−	27 (50)	38 (82)	−	−
1	0	0	−	−	13 (24)	4 (9)	−	−
2	0	0	−	−	3 (6)	1 (3)	−	−
≥3	0	0	−	−	12 (22)	6 (13)	−	−
Median length of stay in ICU (IQR) – days	0 (0–0)	0 (0–0)	−	0.69	0 (0–16)	0 (0–10)	−	0.80
Median length of stay in hospital (IQR) – days related to pancreatitis	0 (0–16)	2 (0–5)	−	0.56	57 (37–90)	41 (22–76)	−	0.09

* Multiple events in the same patient were scored as one outcome.

† 4 patients (of the originally 104 included patients) from the POINTER trial did not consent to participate in this follow-up study and were therefore missing in the total follow-up analysis.

Data are presented as n (%) or median (IQR).

ICU indicates intensive care unit.

### Patients successfully treated with antibiotics only

Of the 17 patients in the postponed-drainage group who survived the initial 6-month follow-up and were successfully treated with antibiotics only, for example, without any interventions, 2 patients did not provide informed consent to this study, leaving 15 patients to be included in these analyses. Of these patients, 14 patients (93%) remained without intervention at the end of long-term follow-up. Ultimately, 14 out of 44 patients (35%) assigned to the postponed-drainage group were successfully treated with antibiotics only in the total follow-up.

### End of long-term follow-up

At the end of long-term follow-up, there were no differences in the new development of exocrine and endocrine pancreatic insufficiency (Table 3). The exocrine and endocrine pancreatic function over time is presented in Supplementary Table S7, Supplemental Digital Content 1, http://links.lww.com/SLA/E733. The quality of life scores, SF-36 physical and mental health scores, at the end of long-term follow-up were also comparable among groups; the physical component scale was 49 (±14) and 43 (±22) (*P*=0·17), whereas the mental component scale was 43 (±8) and 42 (±9) (*P*=0·43) in the immediate- and postponed-drainage group, respectively.

**TABLE 3:** Pancreatic Function and Quality of Life at the End of Long-Term Follow-Up^*^

Endpoint	Immediate drainage (n=42)	Postponed drainage (n=37)	Relative risk (95% CI)	P
Exocrine pancreatic insufficiency				
Enzyme supplement use	18 (43)	13 (35)	1.22 (0.70–2.13)	0.48
Endocrine pancreatic insufficiency	18 (43)	13 (35)	1.22 (0.70–2.13)	0.48
Oral antidiabetics use only	5 (12)	2 (5)	2.20 (0.45–10.68)	0.44
Insulin use only	8 (19)	10 (27)	0.71 (0.31–1.60)	0.40
Oral antidiabetics and insulin use	5 (12)	1 (3)	4.41 (0.54–36.01)	0.21
Quality of Life (SF-36)				
PCS	49 (14)	43 (22)	−	0.17
MCS	43 (8)	42 (9)	−	0.43

* At the end of long-term follow-up, data from questionnaires were obtained from all but one surviving patients (n=79).

Data are presented as n (%) or mean (SD).

The scores of both PCS and MCS range from 0 to 100, with higher scores indicating better quality of life.

PCS indicates Physical Component Scale; MCS, Mental Component Scale.

## DISCUSSION

This long-term follow-up study of the POINTER trial confirms that a postponed-drainage approach for infected necrotizing pancreatitis resulted in fewer interventions, as compared with immediate drainage, and almost a third of these patients were successfully treated with antibiotics only. Postponing or even omitting drainage does not lead to long-term adverse outcomes in patients with infected necrotizing pancreatitis.

In line with previous studies, no benefits of immediate drainage in comparison with delaying intervention were seen.^12,23–26^ Nevertheless, one may argue that a subset of patients still benefits from an immediate approach, as in general, the duration of organ failure impacts clinical outcomes.^27^ A recent pilot randomized controlled trial evaluated the optimal timing of percutaneous drainage in necrotizing pancreatitis with persistent organ failure as the primary indication and reported a beneficial trend for early drainage.^28^ But, the long-term outcomes of both approaches are only evaluated by 1 small nonrandomized study, wherein no difference in regression and recurrence of collections were observed.^26^

The most remarkable benefit of a postponed-drainage approach found in the initial POINTER trial was that 39% of patients assigned to the postponed-drainage group were treated with antibiotics alone (ie, no catheter drainage or other intervention), with 35% of patients surviving the trials’ initial 6-month follow-up.^11^ In the current long-term follow-up study, this benefit continued in 93% of the surviving patients as the intervention was required in 1 initially conservatively treated patient. It is noteworthy that this patient declined cholecystectomy following the initial episode of acute biliary pancreatitis and subsequently developed recurrent acute pancreatitis with infected pancreatic necrosis.

In the total follow-up period, 35% of patients were successfully treated with antibiotics only. It should be pointed out here that the majority of patients did not suffer from (multiple) organ failure at randomization (Supplementary Table S2, Supplemental Digital Content 1, http://links.lww.com/SLA/E733). This is in line with previous studies that have reported similar success rates of antibiotic treatment (range 3% to 39%) in selected patients with infected necrotizing pancreatitis, mostly in patients without organ failure. Future studies will have to confirm the optimal selection criteria for antibiotic treatment, in which procalcitonin should be considered^29^ and determine details of treatment, including aspects of antibiotic stewardship. A prediction model selecting patients for an antibiotics-only approach would be useful and should be developed.

As the results of this study will further enhance the use of antibiotic treatment, efforts to optimize the quality of its use should be made.^30^ A recent Dutch study evaluated antibiotic use and obtained pancreatic cultures of patients with infected necrotizing pancreatitis and found that 48% received inappropriate empirical broad-spectrum antibiotics based on the identified microorganisms.^31^ Another concern about antibiotic (over)use, which in turn has a great impact on antibiotic resistance, is that antibiotics are often not tailored to (FNA-)culture results. Furthermore, the optimal treatment duration for infected necrosis is unknown. We hypothesize that an antibiotic stewardship-driven approach, which includes recommendations on FNA and the timing and duration of antibiotic treatment, will result in similar patient outcomes and health care use as compared with current practice.

During the present long-term follow-up, after the initial 6-month period, necrosectomy was not performed in any patient, meaning that 51% of patients in the immediate-drainage group and 22% of patients in the postponed-drainage group underwent necrosectomy (*P*=0.004) in the total follow-up. This is lower than the 51% to 60% rates of necrosectomy previously reported in patients with infected necrotizing pancreatitis treated with the step-up approach.^18,32^ However, also both these studies stated a negligibly low need for additional necrosectomy after the 6-month follow-up. Another long-term benefit of postponed drainage includes the decreased need for drainage procedures and necrosectomy. The question remains whether postponing drainage through encapsulation of the necrotic collection actually enables a more effective drainage procedure, thereby making multiple procedures and even necrosectomy redundant.^33^

At the end of long-term follow-up, pancreatic function (ie, exocrine and endocrine) did not differ between the 2 groups. Both exocrine and endocrine insufficiency were present in 43% of patients in the immediate-drainage group and 35% in the postponed-drainage group. Previous literature that evaluated late-onset exocrine insufficiency showed similar prevalence rates,^18,32,34^ underlining the importance of monitoring exocrine pancreatic function over time. In our study, the fecal elastase-1 test was only performed in 61% of patients during long-term follow-up. Moreover, we showed that 22% of patients developed endocrine pancreatic insufficiency after the initial 6-month follow-up. It remains unclear, however, how this should be interpreted since we cannot clearly differentiate between post-pancreatitis diabetes and the occurrence of new-onset type 2 diabetes.^35^ Quality of life was similar in both groups. Other long-term follow-up studies in necrotizing pancreatitis patients showed similar quality of life scores, wherein the hypothesis is that over the years, patients adapt to their morbidity, and thereby the quality of life improves when compared with the baseline.^18,36,37^

There are several limitations that need to be taken into account when interpreting the results of this study. First, the sample size was relatively small, although this study the largest follow-up study evaluating both approaches. Second, the long-term follow-up period was not standardized. As a result, the duration of follow-up differed between the first and last randomized patient, ranging from 7 to 2.5 years, respectively. However, in the postponed-drainage group, all first drainage procedures after the initial 6-month follow-up were performed in the first 2 years after randomization with the exception of 1 (Supplementary Table S5, Supplemental Digital Content 1, http://links.lww.com/SLA/E733). In addition, the total follow-up time did not differ between treatment groups. Third, the decision to intervene after the initial 6-month follow-up was not standardized. Nonetheless, the DPSG utilizes a nationwide expert panel,^38^ which helps minimize treatment variation and inequivalent access to specialized care. In cases where the patient showed no improvements with antibiotics, our experts recommended catheter drainage. If drainage had already been performed, further steps, such as a new computed tomography scan and potential drain revision/upgrade or necrosectomy, were advised. Fourth, some data (eg, complications, intervention, hospital stay) were collected retrospectively, which may have led to information bias. Fifth, endocrine and exocrine pancreatic function were pragmatically evaluated based on the use of medication and therefore, do not always reflect the accurate status of pancreatic insufficiency. The main strength is the long-term follow-up of the multicenter randomized POINTER trial in a cohort of patients with infected necrotizing pancreatitis.

## CONCLUSION

Postponed catheter drainage, using antibiotics, may be seen as the preferred approach when treating patients with infected necrotizing pancreatitis. Delaying drainage reduces the number of interventions and offers the opportunity to effectively treat patients with antibiotic treatment only without increased risk for adverse long-term outcomes. The decision to postpone intervention, however, should be individualized and based on the patient’s clinical course and improvement on antibiotics. Further research in this field, including the exact role of antibiotics in the management of infected necrosis, is encouraged.

## Supplementary Material

SUPPLEMENTARY MATERIAL
