# Supplementary material for: Long-Term Outcome of Immediate Versus Postponed Intervention in Patients With Infected Necrotizing Pancreatitis (POINTER): Multicenter Randomized Trial
Source: Ann Surg. 2023 Jul 17;279(4):671–8. doi: 10.1097/SLA.0000000000006001 (PMC10922655; doi:10.1097/SLA.0000000000006001)
Supplement: SUPPLEMENTARY MATERIAL [file sla-279-671-s001.docx]

**SUPPLEMENTARY APPENDIX**

**Supplement to manuscript:**

**Long-term outcome of immediate versus postponed intervention in patients with infected necrotizing pancreatitis (POINTER): multicenter randomized trial**

| Table S1. STROBE checklist | | | |
| --- | --- | --- | --- |
|  | Item No | Recommendation | Page No |
| **Title and abstract** | 1 | (*a*) Indicate the study’s design with a commonly used term in the title or the abstract | Page 1  Page 3 |
|  |  | (*b*) Provide in the abstract an informative and balanced summary of what was done and what was found |  |
| Introduction | | | |
| Background/rationale | 2 | Explain the scientific background and rationale for the investigation being reported | Page 4 |
| Objectives | 3 | State specific objectives, including any prespecified hypotheses | Page 4 |
| Methods | | | |
| Study design | 4 | Present key elements of study design early in the paper | Page 5 |
| Setting | 5 | Describe the setting, locations, and relevant dates, including periods of recruitment, exposure, follow-up, and data collection | Page 5 |
| Participants | 6 | (*a*) Give the eligibility criteria, and the sources and methods of selection of participants. Describe methods of follow-up | Page 5 |
|  |  | (*b*) For matched studies, give matching criteria and number of exposed and unexposed |  |
| Variables | 7 | Clearly define all outcomes, exposures, predictors, potential confounders, and effect modifiers. Give diagnostic criteria, if applicable | Page 6-7 |
| Data sources/ measurement | 8* | For each variable of interest, give sources of data and details of methods of assessment (measurement). Describe comparability of assessment methods if there is more than one group | Page 6-7 |
| Bias | 9 | Describe any efforts to address potential sources of bias | N.A. |
| Study size | 10 | Explain how the study size was arrived at | N.A. |
| Quantitative variables | 11 | Explain how quantitative variables were handled in the analyses. If applicable, describe which groupings were chosen and why | Page 7-8 |
| Statistical methods | 12 | (*a*) Describe all statistical methods, including those used to control for confounding | Page 7-8 |
|  |  | (*b*) Describe any methods used to examine subgroups and interactions |  |
|  |  | (*c*) Explain how missing data were addressed |  |
|  |  | (*d*) If applicable, explain how loss to follow-up was addressed |  |
|  |  | (*e*) Describe any sensitivity analyses |  |
| Results | | |  |
| Participants | 13* | (a) Report numbers of individuals at each stage of study—eg numbers potentially eligible, examined for eligibility, confirmed eligible, included in the study, completing follow-up, and analyzed | Page 9 / Figure 1 |
|  |  | (b) Give reasons for non-participation at each stage |  |
|  |  | (c) Consider use of a flow diagram |  |
| Descriptive data | 14* | (a) Give characteristics of study participants (eg demographic, clinical, social) and information on exposures and potential confounders | Supplementary table S3 |
|  |  | (b) Indicate number of participants with missing data for each variable of interest |  |
|  |  | (c) Summarise follow-up time (eg, average and total amount) |  |
| Outcome data | 15* | Report numbers of outcome events or summary measures over time | Page 9-11 |
| Main results | 16 | (*a*) Give unadjusted estimates and, if applicable, confounder-adjusted estimates and their precision (eg, 95% confidence interval). Make clear which confounders were adjusted for and why they were included | Page 9-11 |
|  |  | (*b*) Report category boundaries when continuous variables were categorized | N.A. |
|  |  | (*c*) If relevant, consider translating estimates of relative risk into absolute risk for a meaningful time period | N.A. |
| Other analyses | 17 | Report other analyses done—eg analyses of subgroups and interactions, and sensitivity analyses | Page 10-11 |
| Discussion |  |  |  |
| Key results | 18 | Summarise key results with reference to study objectives | Page 13 |
| Limitations | 19 | Discuss limitations of the study, taking into account sources of potential bias or imprecision. Discuss both direction and magnitude of any potential bias | Page 15-16 |
| Interpretation | 20 | Give a cautious overall interpretation of results considering objectives, limitations, multiplicity of analyses, results from similar studies, and other relevant evidence | Page 13-16 |
| Generalisability | 21 | Discuss the generalisability (external validity) of the study results | Page 13-16 |
| Other information |  |  |  |
| Funding | 22 | Give the source of funding and the role of the funders for the present study and, if applicable, for the original study on which the present article is based | Page 8 |
| *Give information separately for exposed and unexposed groups. Information on the STROBE Initiative is available at http://www.strobe-statement.org. | | | |

| **Table S2. Definitions of the primary and secondary outcomes** | |
| --- | --- |
| **Outcome** | **Definition** |
| Primary outcome | The primary outcome was a composite of death and major complications. |
| Secondary outcomes |  |
| - Major complications |  |
| New onset organ failure | Organ failure occurring after randomization and not present 24 hours before randomization:  - Pulmonary: a PaO2 < 60 mmHg despite FiO2 30% or the need for mechanical ventilation  - Cardiovascular: a systolic blood pressure < 90 mmHg despite adequate fluid resuscitation or need for vasopressor support  - Renal: a serum creatinine > 177 mmol/L after rehydration or need for hemofiltration or hemodialysis (in case patients already suffered from renal insufficiency before this episode of AP [creatinine > 177 umol/L] this does not count as renal failure) |
| Multiple organ failure | Failure of 2 or more organ systems (i.e. respiratory, cardiovascular or renal) at the same moment. |
| Bleeding requiring intervention | Bleeding requiring surgical, radiologic, or endoscopic intervention. |
| Perforation of a visceral organ requiring intervention | Perforation requiring surgical, radiologic, or endoscopic intervention. |
| Enterocutaneous fistula requiring intervention | Secretion of fecal material from a percutaneous drain or drainage canal after removal of drains or from a surgical wound, either from small or large bowel; confirmed by imaging or during surgery. |
| - Other outcomes |  |
| Incisional hernia | Incisional hernia is defined as full-thickness discontinuity in abdominal wall and bulging of abdominal contents, with or without obstruction. |
| Pancreaticocutaneous fistula | Output through a percutaneous drain or drainage canal after removal of drains from a surgical wound, or any measurable volume of fluid with an amylase content >3 times the serum amylase level. |
| Wound infection | A superficial incisional SSI (surgical site infection) and must meet the following criterion: infection occurs within 30 days after the operative procedure and involves only skin and subcutaneous tissue of the incision and the patient has at least 1 of the following:  - Purulent drainage from the superficial/deep incision but not from the organ/space component of the surgical site  - Organisms isolated from an aseptically obtained culture of fluid or tissue from the superficial incision  - At least 1 of the following signs or symptoms of infection: pain or tenderness, localized swelling, redness, or heat, and superficial incision is deliberately opened by surgeon and is culture positive or not cultured. A culture-negative finding does not meet this criterion  - An abscess or other evidence of infection involving the deep incision is found on direct examination, during reoperation, or by histopathological or radiologic examination  - Diagnosis of superficial/deep incisional SSI by the surgeon or attending physician |
| Exocrine pancreatic insufficiency | Oral pancreatic-enzyme supplementation required to treat clinical symptoms of steatorrhea; this requirement was not present before onset of pancreatitis. |
| Endocrine pancreatic insufficiency | The need for insulin or oral-diabetic drugs; this requirement was not present before onset of pancreatitis. |
| Recurrent acute pancreatitis | Recurrence of acute pancreatitis is defined as a new episode of acute pancreatitis, as defined by the 2012 Revised Atlanta criteria, after complete resolution of all symptoms associated with the previous acute pancreatitis episode. |
| Chronic pancreatitis | Defined according to the M-ANNHEIM criteria. |

| **Table S3. Baseline characteristics of the POINTER trial** | | |
| --- | --- | --- |
| **Characteristics** | **Immediate Drainage**  **(*n* = 55)** | **Postponed Drainage**  **(*n* = 49)** |
| Age (yr) | 60 (14) | 59 (11) |
| Male sex | 32 (58) | 32 (65) |
| Cause of pancreatitis |  |  |
| Gallstones | 36 (65) | 29 (59) |
| Alcohol abuse | 8 (15) | 7 (14) |
| Disease severity |  |  |
| Admitted to intensive care unit | 15 (27) | 13 (27) |
| SIRS | 47 (85) | 40 (82) |
| Organ failure | 13 (24) | 8 (16) |
| Multiple organ failure | 8 (15) | 6 (12) |
| CT severity index^a^ | 7 ± 2 | 6 ± 2 |
| Extent of pancreatic necrosis |  |  |
| <30% | 35 (64) | 33 (68) |
| 30-50% | 8 (15) | 7 (14) |
| >50% | 12 (22) | 9 (18) |
| Encapsulation of necrosis |  |  |
| Not encapuslated | 6 (11) | 8 (16) |
| Medium encapsulated | 16 (29) | 19 (39) |
| Largely encapsulated | 19 (35) | 11 (22) |
| Fully encapsulated | 14 (25) | 11 (22) |
| Diagnosis of infected necrosis |  |  |
| Gas configuration | 20 (36) | 16 (33) |
| Positive fine needle aspiration | 6 (11) | 11 (22) |
| Suspected clinically | 29 (53) | 22 (45) |
| Onset of symptoms to diagnosis of necrotising pancreatitis/ necrotic collection (days) | 8 ± 8 | 9 ± 7 |
| Onset of symptoms to diagnosis of infected necrosis (days) | 21 ± 6 | 19 ± 7 |
| Data are presented as no. (%) or mean (SD). CT = computed tomography, SIRS = Systemic Inflammatory Response Syndrome. ^a^Data were derived from the contrast-enhanced CT performed before randomization. Scores may range from 0 to 10, with higher scores indicating more extensive pancreatic and peripancreatic necrosis. | | |

| **Table S4. All-cause mortality after the initial 6-month follow-up per individual patient** | | | | |
| --- | --- | --- | --- | --- |
| **Immediate-drainage** | **Cause of death** | **Age** | **Pancreatitis related** | **Time (months)^a^** |
| 1 | Obstructive shock of unknown cause | 72 | No | 8 |
| 2 | Multiple causes not related to pancreatitis | 69 | No | 9 |
| 3 | Fistula of the gastrointestinal-tract (patient requested life-sustaining treatment withdrawal) | 70 | Yes | 10 |
| 4 | Infected pancreatic necrosis in combination with COVID-19 infection (patient requested life-sustaining treatment withdrawal) | 57 | Yes | 48 |
| **Postponed-drainage** | **Cause of death** | **Age** | **Pancreatitis related** | **Time (months)^a^** |
| 1 | Cholangiocarcinoma | 57 | No | 26 |
| 2 | Gastric cancer | 75 | No | 44 |
| 3 | Respiratory failure of unknown cause | 56 | No | 57 |
| 4 | Lung cancer | 74 | No | 62 |
| ^a^Time between randomization and date of death. | | | | |

| **Table S5. Drainage procedures after the initial 6-month follow-up per individual patient** | | | |
| --- | --- | --- | --- |
| **Immediate-drainage** | **Type of drainage** | **Indication** | **Time (months)^a^** |
| 1 | - PCD (5x) | Persistent pancreatic fluid collection | 7 |
| 2 | - PCD | Recurrent pancreatic fluid collection (recurrent acute pancreatitis) | 49 |
| 3 | - PCD | Persistent pancreatic fluid collection (disconnected pancreatic duct) | 20 |
| 4 | - ETD | Recurrent pancreatic fluid collection | 7 |
| 5 | - ETD | Recurrent pancreatic fluid collection (disconnected pancreatic duct) | 9 |
| 6 | - PCD | Recurrent pancreatic fluid collection (disconnected pancreatic duct) | 23 |
| 7 | - ETD | Persistent pancreatic fluid collection | 15 |
| **Postponed-drainage** | **Type of drainage** | **Indication** | **Time (months)^a^** |
| 1 | - PCD | Persistent pancreatic fluid collection | 11 |
| 2 | - ETD | Recurrent pancreatic fluid collection (recurrent acute pancreatitis) | 22 |
| 3 | - ETD  - PCD (20x) | Recurrent infected necrotic collections (recurrent acute pancreatitis) | 19 |
| Patients per group who required (additional) drainage procedures after the initial 6-months follow-up. ETD = endoscopic transluminal drainage. PCD = percutaneous catheter drainage. **^a^**Time between randomisation and date of first drainage procedure after the initial 6-months follow-up. | | | |

| **Table S6. Sensitivity analyses: Primary outcome and interventions in patients whom diagnosis was based on clinical suspicion for infected necrosis^a^** | | | | | | | | |
| --- | --- | --- | --- | --- | --- | --- | --- | --- |
|  | **New events after the initial 6-month follow-up**  (excluding events as initially reported in the POINTER trial) | | | | **Total follow-up^b^**  (Time between randomization and the end of long-term follow-up) | | | |
| **Outcome** | **Immediate**  **Drainage**  **(*n* = 24/47)** | **Postponed Drainage**  **(*n* = 18/41)** | **Relative**  **risk**  **(95% CI)** | **P-value** | **Immediate**  **Drainage**  **(*n* = 29/54)** | **Postponed Drainage**  **(*n* = 20/46)** | **Relative**  **risk**  **(95% CI)** | **P-value** |
| **Primary outcome** | | | | | | | | |
| Major complications or death – no. (%) | 3 (13) | 3 (17) | 0.75 (0.17-3.29) | 1.00 | 15 (52) | 9 (45) | 1.15 (0.63-2.09) | 0.64 |
| **Interventions** | | | | | | | | |
| Catheter Drainage – no. (%) | 4 (17) | 3 (17) | 1.00 (0.26-3.92) | 1.00 | 29 (100) | 14 (70) | 1.43 (1.07-1.90) | 0.00 |
| Necrosectomy – no. (%) | 0 | 0 | - | - | 18 (62) | 4 (20) | 3.10 (1.24-7.80) | 0.00 |
| Median total surgical, endoscopic, and radiologic interventions for infected necrosis (IQR) – no. | 0 (0-0) | 0 (0-0) | - | 0.94 | 4 (2-7) | 2 (0-5) | - | 0.06 |
| Median total drainage procedures (IQR) - no. | 0 (0-0) | 0 (0-0) | - | 0.94 | 2 (1-5) | 1 (0-4) | - | 0.07 |
| No. of drainage procedures (%) – no. of patients (%) |  |  |  |  |  |  |  |  |
| 0 | 20 (83) | 15 (83) | - | - | 0 | 6 (30) | - | - |
| 1 | 4 (17) | 2 (11) | - | - | 13 (45) | 7 (35) | - | - |
| 2 | 0 | 0 | - | - | 2 (7) | 0 | - | - |
| ≥3 | 0 | 1 (1) | - | - | 14 (48) | 7 (35) | - | - |
| Median total necrosectomies (IQR) – no. | 0 (0-0) | 0 (0-0) | - | - | 1 (0-3) | 0 (0-0) | - | 0.03 |
| No. of necrosectomies – no. of patients (%) |  |  |  |  |  |  |  |  |
| 0 | 24 (100) | 18 (100) | - | - | 11 (38) | 16 (80) | - | - |
| 1 | 0 | 0 | - | - | 6 (21) | 0 | - | - |
| 2 | 0 | 0 | - | - | 3 (10) | 0 | - | - |
| ≥3 | 0 | 0 | - | - | 9 (31) | 4 (20) | - | - |
| Data are presented as no. (%) or median (IQR). ^a^Multiple events in the same patient were scored as one outcome. ^b^4 patients (of the originally 104 included patients) from the POINTER trial did not consent to participate in this follow-up study and were therefore missing in the total follow-up analysis. ICU = intensive care unit. | | | | | | | | |

| **Table S7. Exocrine and endocrine pancreatic function over time^a^** | | | |
| --- | --- | --- | --- |
| **Endpoint** | **Total**  **(n=79)** | **Immediate**  **Drainage**  **(*n* = 42)** | **Postponed**  **Drainage**  **(*n* = 37)** |
| Exocrine pancreatic insufficiency^b^ |  |  |  |
| No | 42 (53) | 23 (55) | 19 (51) |
| Recovered | 6 (8) | 1 (2) | 5 (14) |
| Persistent | 25 (32) | 15 (36) | 10 (27) |
| New-onset | 6 (8) | 3 (7) | 3 (8) |
| Endocrine pancreatic insufficiency^c^ |  |  |  |
| No | 44 (56) | 23 (55) | 21 (57) |
| Recovered | 4 (5) | 1 (2) | 3 (8) |
| Persistent | 14 (18) | 7 (18) | 7 (19) |
| New-onset | 17 (22) | 11 (26) | 6 (16) |
| Data are presented as no. (%). ^a^Pancreatic function at the end of long-term follow-up compared to the pancreatic function at 6-month follow-up. Data from questionnaires were obtained from all but one surviving patients (n=79).^b^ Defined as pancreatic enzyme use. ^C^Defined as diabetes medication use. | | | |
